# Digital and Precision Technologies in Dairy Cattle Farming: A Bibliometric Analysis

**DOI:** 10.3390/ani14121832

**Published:** 2024-06-20

**Authors:** Franck Morais de Oliveira, Gabriel Araújo e Silva Ferraz, Ana Luíza Guimarães André, Lucas Santos Santana, Tomas Norton, Patrícia Ferreira Ponciano Ferraz

**Affiliations:** 1 Department of Agricultural Engineering, School of Engineering, Federal University of Lavras (UFLA), Lavras 37200-900, Brazil; franck.oliveira1@estudante.ufla.br (F.M.d.O.); patricia.ponciano@ufla.br (P.F.P.F.); 2 Department of Animal Science, Federal University of Lavras (UFLA), Lavras 37200-900, Brazil; ana.andre@estudante.ufla.br; 3 Department of Agricultural and Environmental Engineering (EEA), Institute of Agricultural Sciences (ICA), Federal University of Vales Jequitinhonha and Mucuri—Campus Unaí, Avenida Universitária, nº 1.000, B. Universitários, Unai 38610-000, Brazil; lucas.unemat@hotmail.com; 4 M3-BIORES-Measure, Model & Manage Bioresponses, KU Leuven, Kasteelpark Arenberg 30, B-3001 Leuven, Belgium; tomas.norton@kuleuven.be

**Keywords:** precision livestock farming, dairy cows, technologies, bibliometric review

## Abstract

**Simple Summary:**

Technological advancements have revolutionized dairy cattle management through digital and precision approaches. This study conducts a bibliometric analysis of these technologies, identifying emerging patterns, research themes, and author collaborations. It reveals top journals of interest and emerging technologies such as machine learning and computer vision. These tools are crucial for decisions enhancing health and efficiency in milk production, promoting more sustainable practices. It highlights the evolution of precision livestock farming and introduces digital livestock farming, demonstrating how advanced digital tools transform dairy herd management. This shift not only boosts productivity but also redefines cattle management, emphasizing its impact on the sustainability and efficiency of milk production.

**Abstract:**

The advancement of technology has significantly transformed the livestock landscape, particularly in the management of dairy cattle, through the incorporation of digital and precision approaches. This study presents a bibliometric analysis focused on these technologies involving dairy farming to explore and map the extent of research in the scientific literature. Through this review, it was possible to investigate academic production related to digital and precision livestock farming and identify emerging patterns, main research themes, and author collaborations. To carry out this investigation in the literature, the entire timeline was considered, finding works from 2008 to November 2023 in the scientific databases Scopus and Web of Science. Next, the Bibliometrix (version 4.1.3) package in R (version 4.3.1) and its Biblioshiny software extension (version 4.1.3) were used as a graphical interface, in addition to the VOSviewer (version 1.6.19) software, focusing on filtering and creating graphs and thematic maps to analyze the temporal evolution of 198 works identified and classified for this research. The results indicate that the main journals of interest for publications with identified affiliations are “Computers and Electronics in Agriculture” and “Journal of Dairy Science”. It has been observed that the authors focus on emerging technologies such as machine learning, deep learning, and computer vision for behavioral monitoring, dairy cattle identification, and management of thermal stress in these animals. These technologies are crucial for making decisions that enhance health and efficiency in milk production, contributing to more sustainable practices. This work highlights the evolution of precision livestock farming and introduces the concept of digital livestock farming, demonstrating how the adoption of advanced digital tools can transform dairy herd management. Digital livestock farming not only boosts productivity but also redefines cattle management through technological innovations, emphasizing the significant impact of these trends on the sustainability and efficiency of dairy production.

## 1. Introduction

Digital and Precision Livestock Farming emerges as a dynamic and innovative field in the livestock sector, especially in dairy farming. Driven by advanced technologies, this field seeks to improve the management, monitoring, and performance of herds, representing a transformation in the conventional approach to livestock farming. Automation in livestock production has become imperative, given the increase in the size of farms and labor costs, reducing the time needed to monitor animals and offering a more efficient approach [1].

The concept of precision livestock farming (PLF), also known as precision livestock production (PLP), expands the principles of precision agriculture by incorporating elements from agricultural ecosystems with a special focus on animal interactions, seeking to optimize production systems to make them more efficient and environmentally sustainable [2]. Through the implementation of these technological solutions, producers have the ability to access detailed information about their herds, enabling more efficient and proactive management.

On the other hand, digital livestock farming represents a subsequent evolution, specifically by interconnecting modern livestock information technologies such as big data analysis, computer vision, artificial intelligence, machine learning, and deep learning to further improve the efficiency and management of herds. While precision farming involves the use of sensors and technologies to collect detailed data about animals and the environment, digital farming goes further, incorporating these technologies in real time, taking producers into a new era of agricultural management. With the interconnection of these technologies, digital livestock farming allows producers to access more comprehensive information about herd behavior, health, and performance. This innovative approach not only makes it easier to identify complex patterns but also enables the automation of decision-making processes.

In the first theme, an example of precision livestock farming relates to the investigation of heat stress, a critical challenge in modern livestock farming that impacts the well-being, health, and production of cows. In [3], the impact of heat stress on rumination, feeding, and locomotor activity in cows during calving, especially those diagnosed with subclinical ketosis in the first 30 days postpartum, was examined. Furthermore, global consumption of animal products has been leveling off in first-world countries but is increasing rapidly in developing countries, driving the need to increase agricultural production and adopt technological innovations, including precision farming [4]. Other research has explored technologies such as collars with accelerometer-based sensors to record behaviors and breathing [5], developing machine learning solutions to monitor behavioral and physiological changes [6], as well as nose device electronic systems for the detection of volatile organic compounds [7].

In the context of dairy farming, Artificial Intelligence technology, together with computer vision algorithms such as YOLOv3, has been applied to characterize the thermal environment inside compost barns and evaluate the standing and lying behavior of cows [8]. Other innovations include the use of YOLO V5s for real-time individual identification of dairy cows [9] and automated sensors to detect changes in health indicators [10], highlighting the potential of these technologies for more effective monitoring and management of the herd. These technologies, with their advantages of being contactless, stress-free, low cost, and high yield, have a broad perspective of application in animal production [11].

The analysis of feed consumption, as a crucial indicator of production performance and the risk of diseases in dairy cows [12], and the measurement of animal dimensions as essential indicators to monitor growth rate, food efficiency, and health status [13], highlight the importance of assessing body condition as an indicator of cattle health. Advances in the quantitative analysis of three-dimensional shapes [14] promote the development of smart agriculture.

Therefore, when some references to these technologies are presented, precision livestock farming is understood as the application of Information and Computer Technology (ICT) for monitoring and managing animals [15]. The precision phase involves the implementation of sensors to measure parameters in the environment and animals, providing essential data for livestock management. At this stage, information collection is improved but does not yet incorporate advanced analyses or more complex technologies. This concept incorporates digital technologies from sensors integrated into wearable devices, GPS tracking, and traceability systems.

The incorporation of advanced technologies, with artificial intelligence algorithms, in digital livestock farming provides a deeper understanding of the individual behaviors and needs of dairy cattle. By proactively predicting trends, optimizing feed, and monitoring health, digital livestock farming not only improves operational efficiency but also contributes to sustainable practices. Notable examples include automated monitoring systems to improve dairy production [15], the use of a trained neural network to identify the basic behaviors of a dairy cow in an environment [16], and the application of computer vision with a convolutional neural network for detailed analysis of the body condition of dairy cattle [17]. This evolution in farm management, which goes beyond precision farming, empowers producers to meet contemporary farming challenges with a digitally advanced, data-driven approach, resulting in highly specialized and personalized strategies to optimize dairy production.

In summary, while precision livestock farming prioritizes efficiency in data collection and monitoring, digital livestock farming stands out for its more advanced approach, employing complex data analysis and specialized technologies, such as machine learning and computer vision. This differentiation allows for highly personalized decisions in the management of dairy cattle, resulting in more precise and adapted strategies to optimize production. Furthermore, the increasing complexity and interconnectedness of modern agricultural systems require more advanced and precise approaches to ensure the efficiency and sustainability of milk production. Therefore, understanding the evolution and impact of digital and precision technologies in dairy farming is fundamental not only for producers but also for researchers, policymakers, and professionals in the sector who seek to promote more innovative and sustainable agricultural practices.

In this context, digital and precision dairy farming gains relevance not only as a response to current challenges but as an innovative approach to improving production, sustainability, and efficiency in livestock farming. Thus, the objective of this study was to evaluate the evolution of publications on digital and precision technologies in livestock farming, highlighting advances, trends, and contributions of authors in the area, identifying the main journals, authors, countries, and relevant organizations that are associated with the filtered publications by the keywords defined and most used in publications, and finally, identifying trends regarding these technologies through this bibliometric analysis.

## 2. Materials and Methods

**Figure 1:**
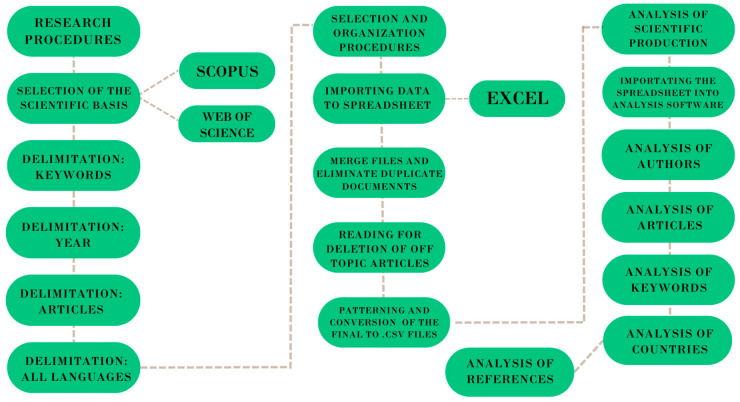
Systematization of steps for bibliometric analysis.

The development of scientific research examining precision and digital livestock was evaluated through bibliometric analysis, following a flowchart (Figure 1). Bibliometric analysis provides a broader observation of science and technology through scientific productions in a database. Specifically, bibliometric systematics can be divided into data retrieval, preprocessing, network extraction, normalization, mapping, and analysis [18,19].

### 2.1. Research Procedure

Before the bibliographic analysis is started, understanding the topic that will be addressed is essential. Specifically, the topic needs to be filtered, and the most relevant parts should be selected. Therefore, some points, such as the time interval of the searches, the area and subarea, the language, and the type of file that need to be included in the study, should be delimited before database searches are conducted [20].

The scientific databases that were selected for finding articles were Scopus and the Web of Science because they are relevant in bibliometric studies and represent the largest article databases worldwide [21,22]. Additionally, these databases present quality indicators represented by the number of citations (JCR) and the H index; therefore, they are more often used in bibliometric studies [23].

After the databases to be explored were delimited, the next step involved selecting representative keywords related to the research theme. This research aimed to develop a bibliometric analysis of precision and digital livestock, focusing on dairy cattle. In this step, words that promoted the generalization of the results were eliminated. Additionally, enough key terms were chosen so that related search topics would not be restricted or included [24]. This is important for avoiding trends that can affect the results [25].

Therefore, to conduct searches within the scientific databases, it was necessary to organize the keywords into three distinct groups. The first set of words included “precision dairy farming”, “precision livestock”, “digital livestock”, and “smart farming”, which specified that dairy farming was the area being addressed. The second group of words clustered the terms that specified the type of animal addressed in the study on precision and digital livestock; these included “dairy cattle” and “dairy cow”. Thus, the chosen terms were “smart cattle”, “big data”, “system”, “information* transmission”, “data transmission”, “transmission technologies”, “remote sensing”, “environment”, “electronic”, “monitoring”, “automat”, “wearable”, “wireless”, “image analysis”, “sound analysis”, “innovation”, “computer vision”, “IOT”, “artificial intelligence”, “algorithm”, “sensor”, “neural network”, “deep learning”, “machine learning”, “GPS”, “spatial variability”, “tracking”, “behaviour”, “identification”, and “traceability”.

In the Scopus database, in the document search tab, we selected the article title, abstract, and Keywords. In the Web of Science, the search was performed in its main collection, with the selection of the documents tab and, in the search engine, the topic tab [26]. Some search operations were conducted to improve its accuracy, and asterisks were used for those terms that could be found both in plural and singular forms. Additionally, with the aim of avoiding the repetition of terms within the sets, parentheses corresponding to the grouping terms and the OR term were used to find records that contained words from the same grouping or from others. Both in Scopus and in the Web of Science, the documents were limited to only articles from 2008 to November 2023. The year 2008 was chosen because it was the first occurrence of a research paper with this set of keywords.

### 2.2. Selection and Organization Procedures

The data selection and organization procedure involved reviewing the information obtained. After the databases were searched, a total of 405 documents were found, with 194 articles obtained from the Scopus database and 211 articles from the Web of Science. These documents were then subjected to a filtering process to eliminate conference materials, review articles, and duplicate works (168 articles removed), and finally, works that did not correspond to the topic were also removed (39 articles removed). The works considered not to correspond to the theme were those in which, although they contained some keywords related to the theme of this research, “cows/dairy cows/dairy cattle” was mentioned only incidentally, while the main focus of the study was on other animals or other aspects of animal production. Thus, 198 documents were selected for this bibliometric study.

### 2.3. Bibliometric Mapping and Clustering

The selected documents were analyzed in R software version 4.3.1 (R Development Core Team, R project, Vienna, Austria) using the Bibliometrix library version 4.1.3 [27] of R software and in VOSviewer version 1.6.19. Bibliometrix is a free library of R software that was developed to perform bibliometric analysis and cocitations of scientific publications [27]. With this library, it was possible to perform a quantitative and qualitative analysis of bibliographic data; in addition, it was possible to identify patterns and trends in the scientific production of the analyzed articles. Among its functionalities, this facilitates the extraction of data from databases, where Scopus and Web Science databases were used for this work, and the performance of several statistical packages for the creation of graphs and thematic maps to visualize the results. With Bibliometrix, it is possible to evaluate scientific production in different areas of knowledge, identify the main areas of research, and follow the evolution of scientific knowledge over time. Additionally, Biblioshiny [28], which is an extension of the Bibliometrix package, was used. This application facilitates the creation of an interface for viewing and exploring the results of bibliometric analysis. This function creates a web application with a particular IP address, which can be run locally. With Biblioshiny, it is possible to create interactive dashboards with graphs, tables, and maps that allow the user to dynamically explore data, interact with variables, and discover relevant insights from the analyzed data.

The other software used in this procedure was VOSviewer. This software can be used to create and visualize bibliometric maps and can be used for mapping authors or journals through cocitation data, keywords, or co-occurrence [29,30,31]. Therefore, this software was used to map the most relevant affiliations, author’s cocitation networks, co-occurrence of keywords, and collaboration between authors.

## 3. Results and Discussion

**Figure 2:**
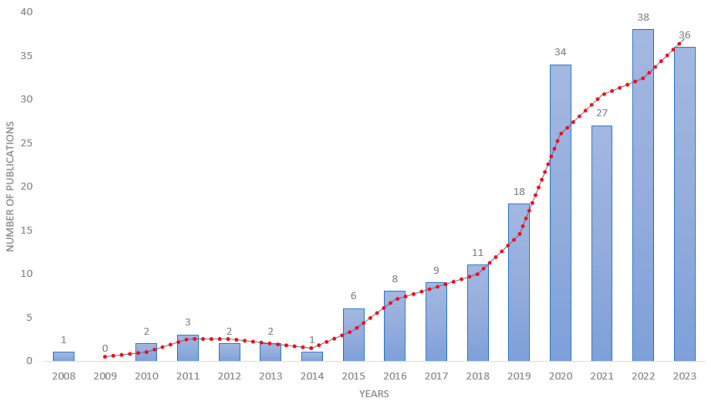
Temporal evolution of the research publications on digital and precision livestock from 2008 to November 2023.

The bibliometric analysis of the chosen criteria revealed 198 scientific articles related to digital and precision livestock published between 2008 and November 2023. The temporal evolution of these publications is presented in Figure 2. The annual growth rate is 26.99%. These papers came from 59 different sources and were written by 830 authors, with an average of 5.96 coauthors per document. No paper was published by a single author. Additionally, the analyses revealed that the average number of citations per document was 18.21 and that the papers referenced 7174 scientific works.

In 2008, only one research article following the research criteria on digital and precision livestock was published. Ref. [1] conducted a study involving two different systems to detect lameness in dairy cows using sensors, with the aim of developing an automated method independent of an observer for this diagnosis. The first system has four scales that measure the weight of the animal’s legs during robotic milking, while the second system works from dynamic forces performed by a fit electromechanical film mat and is installed in an environment easily accessible by the animals.

In 2010, two publications from the same first author were found. Ref. [32] developed a study to compare two image analysis methodologies to describe the behavior of dairy cows confined in confinement barns. The first methodology analyzed images generated directly by the computer and captured by six cameras housed in the facilities, while the second methodology was based on images provided by the software. However, for both methods, no marked differences were found regarding the animals’ activities. In the same year, the author published a second work addressing the time spent at rest by Holstein-Fresian cows influenced by milk production, lactation stage, and body condition score from an automated sensor to monitor the animal’s activities [33].

These initial papers were crucial in paving the way for further exploration of this topic. There was significant growth in the number of publications starting in 2019, with a notable peak in 2020, totalling thirty-four publications covering various topics related to dairy cattle integrated with digital and precision technologies. However, in 2021, there was a decline in the number of publications potentially attributed to the COVID-19 pandemic, but there was a resurgence of publications in 2022, and positive development persisted until November 2023. The evolution of publications in this field since 2019 can be attributed to the growing development of computational processing, which has facilitated the development of novel approaches for data analysis, sensor creation, and algorithms.

### 3.1. Relevant Publications and Characteristics of Papers

The effects of publications are strongly associated with the number of times an article from a given journal is cited [34]. Thus, for this study, the ten most relevant articles were ranked according to the number of citations, as shown in Table 1.

The most cited research article in the last fifteen years was published by [35], with 166 citations. The authors sought to classify the biologically important behaviors of dairy cows using a decision tree algorithm with information provided by an accelerometer. Thus, they emphasized the application of the algorithm as a real-time behavioral monitoring system adopted in daily farm life, with the aim of automatically monitoring and recording the health and well-being conditions of the animals.

The second author with the highest number of citations conducted a study on the dairy industry and its need to adapt to the current market by becoming more resource-efficient, environmentally friendly, transparent, and secure. They introduced a platform oriented towards implementing Internet of Things (IoT) techniques, edge computing (EC), artificial intelligence (AI), and blockchain within the smart farming environment, designed to monitor the state of dairy cattle and feed grain in real time, as well as to ensure the traceability and sustainability of the various processes involved in production, and they found it to be a good solution [36].

The paper developed by [4], who was listed as the third most cited author, contributed significantly to the field with the research titled “Smart Animal Agriculture: Application of Real-Time Sensors to Improve Animal Well-Being and Production”. Published in collaboration with Marcella Guarino, Jeffrey Bewley, and Matti Pastell, this study addresses the evolving landscape of animal product consumption, emphasizing the rising demand in developing countries. The abstract emphasizes the need for agriculture to adapt and increase output, leading to enhanced automation, technological innovation, sustainable farming practices, and the implementation of precision livestock farming (PLF) applications. The authors highlight the growing prevalence of early indicators for medical issues, using sensors to alert cattle farmers promptly about individual animals requiring special care. Wearable technologies, predominantly sensors, dominate the market, with a foreseeable shift toward one sensor per herd, flock, or school in less-value-per-animal systems such as sheep, goats, pigs, poultry, and fish. Despite the immense amount of data generated by PLF sensors, the absence of standards for sensor-generated data sharing currently limits the full utilization of commercial sensors. The authors emphasize the pivotal role of technologies that provide accurate data in enhancing well-managed farms and stress the critical need for developing methods to translate data into actionable solutions.

The scientific monitoring device developed by [37] is a new tool for automated measurements of rumination data and other biological behaviors of dairy cows housed in a barn. This device utilizes a nasal band sensor based on generic algorithms, fulfilling the authors’ expected objective as a new data source for animal health and welfare.

A three-dimensional algorithm involving image processing and regression mechanisms to aid in the identification of the body condition score (BCS) using a low-cost three-dimensional Kinect camera that achieved good repeatability was presented by [38].

The sixth most cited work is that by [33]. Their work presented the use of accelerometer technology to monitor the behavior of lactating cows with varying body conditions and milk production scores. The authors calculated an average BCS of 3 weeks for each cow, and at the beginning of the experiment, 84 cows were selected; however, for the final analysis, 77 cows were considered, excluding cows that presented clinical lameness before or during the observation period, who were classified into three BCS categories and two lactation categories. The cows’ behaviors were observed for 408 days. The mixed model was adjusted to describe the average daily hours spent lying down, and the results showed that although an increase in sleeping hours was observed with increasing BCS, after the other factors were included in the mixed model, BCS did not significantly impact rest time. Therefore, these management factors that affect resting time must be investigated further using new technologies with a larger number of cows under analysis, which will help dairy owners better manage their facilities and cow behaviors.

The work developed by [39] involves the design and implementation of a computer vision system to measure individual cow feed consumption based on deep convolutional neural network (CNN) models and a low-cost RGB-D (red, green, blue, depth) camera. The authors positioned this camera above the feeding area in an open warehouse, and consumption was estimated by combining information from RGB images and depth. The analysis showed that the quantity and diversity of data are important for training these models and that better data results were obtained for a model with high diversity than for a model trained with homogeneous data. Additionally, the authors state that the analysis of training the model based on RGB-D data presents better results than the model based on depth data only, suggesting that low-cost cameras for measuring individual feed consumption have good potential.

Following the theme of animal behavior, but in different kinds of housing systems, [40] applied a methodology based on computer vision in dairy cows housed in free-stall barns using the Viola–Jones algorithm to analyze feeding behavior and animal behavior, such as standing and lying down. The system proved to be efficient for measuring the behavioral indices of the animals in real time.

In ninth place, [41] presents the physiological work entitled “Behavioral and physiological changes around estrus events identified using multiple automated monitoring technologies”. This work had two objectives: to describe the changes related to the heat of 35 cows in three groups between January and June 2013 in the parameters recorded with five automated monitoring technologies and to explore the potential of stress detection with machine learning techniques using automatically collected data. The results showed that the accuracy of the machine learning algorithms for all technologies ranged from 91.0 to 100.0%, and based on these results, these techniques have the potential to be applied to automatically collect technological data for heat detection.

In tenth place in this ranking, an author conducted image analysis to individually identify and monitor the feeding behavior of 17 dairy cows using convolutional neural networks. One neural network was used to detect the presence of a cow in the feeding area, and another network was used to determine the cow’s position in front of the feeder, either standing or feeding. A third neural network checked for the availability of food in the feeder and, if present, recognized the food category. The final neural network was designed for individual dairy cow identification. The authors also explored the contribution of a neural network coupled to a support vector machine (SVM) and the combination of multiple networks. The results showed that the methods used produce high scores at each step of the algorithm [42].

### 3.2. Most Influential Journals

The journals were ranked by their worldwide relevance (Table 2). The journals showed similarities in terms of the themes, which are linked to agricultural sciences and technology. The journal “Computers and Electronics in Agriculture” was ranked as the most important according to the number of documents available in the research database and by the highest number of citations. Its influence can be relevant to its breadth, covering a wide range of topics to the digital livestock community. Table 1 shows that this journal has one of the most cited papers [37]. According to [43], this journal is among the most significant journals in the agricultural field.

The “Journal of Dairy Science” appears to be the second most important in this ranking; it has 715 citations and 23 documents published in this evaluated period, and three of the most cited articles were also presented [38,39,41] (Table 1). Its high number of citations suggests that the published research has an impact and is frequently referenced by the scientific community.

Detailed information about each journal was carefully extracted from the respective official websites, providing a comprehensive overview of each publication’s focus areas and specializations. The journal Computers and Electronics in Agriculture focuses on advances in hardware, software, and electronics applied to agriculture, including agronomy, horticulture, forestry, aquaculture, and livestock farming, and offers a crucial technological perspective on digital and precision livestock farming. Articles related to the integration of computational technologies in the management of herds and agricultural environments can be found in this journal, so the largest number of citations and documents for this research were found in this journal, as many keywords were entered as keywords in the search of the technologies applied to livestock farming [44].

As the premier dairy research journal, the Journal of Dairy Science addresses biochemical, genetic, and public health issues related to dairy production. This journal is essential for understanding how precision farming influences milk production and general practices in the dairy industry [45]. This research focused on dairy cattle, and major researchers have sought to use precision technologies to manage and improve dairy productivity activities.

Overall, these key journals play essential roles in understanding and advancing digital and precision livestock farming. These publications offer a comprehensive and interdisciplinary view, covering everything from the development and application of computational and electronic technologies in agriculture, such as computers and electronics in agriculture [44] and biosystems engineering [46], to more specific issues, such as biochemistry and genetics in milk production, highlighted by the Journal of Dairy Science [45] and Journal of Dairy Research [51]. The journal Animals [47] covers several animal-related disciplines, contributing to an understanding of animal welfare ethics in precision farming, while the journal Animal [48] focuses on innovative science related to animal production, including genetics and production systems. Animal Biotelemetry [49] is notable because it provides insights into telemetric techniques applied to agricultural environments, and Ad Hoc Networks [50] contributes knowledge about sensor networks that is relevant for animal monitoring. Taken together, these journals provide a comprehensive view of the technologies, practices, and innovations shaping precision livestock farming.

### 3.3. Author Publications

**Figure 3:**
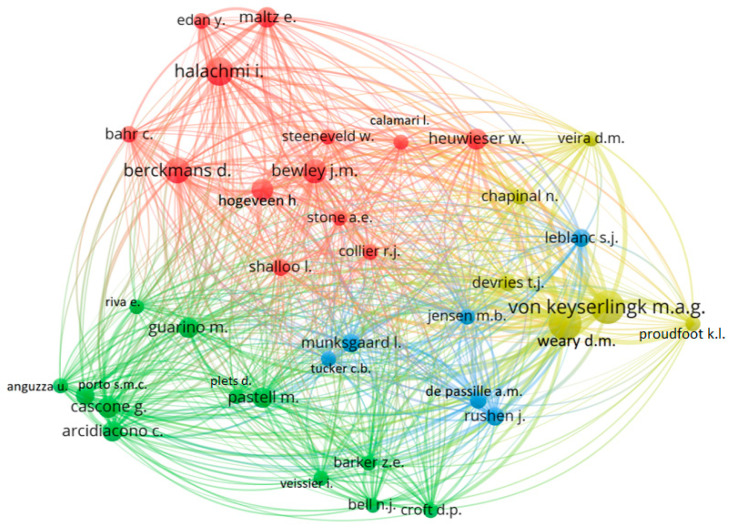
Scientific mapping of the cocitation of most relevant authors in digital and precision livestock research.

To perform a comprehensive analysis of the cocitation network among influential authors in digital and precision livestock research, it is essential to examine the interconnections between their scholarly contributions. Cocitation analysis facilitates the identification of significant patterns and relationships between the most relevant authors, highlighting the interconnectivity of their work and the joint influence exerted on the field. By these mapping connections, significant insights can be gained into the trends, intellectual partnerships, and areas of focus that shape the development of digital and precision livestock farming. Figure 3 shows a cocitation map of the most relevant authors in digital and precision livestock research. The authors were considered to have at least 30 citations in the works of this research, which made it possible to classify 37 authors.

The cocitation map illustrates the scientific network of a study based on the frequency with which two articles are cited together by a third document [52]. Author cocitation refers to the frequency with which two authors are cited together in a third document. In simple terms, it is a measure that illustrates the connection or association between two authors based on how many times their works are referenced simultaneously by other authors in their publications.

In an author cocitation map, circles represent authors, and the lines between them indicate the frequency with which they are cited together in academic or scientific works. The size of these circles is usually related to their importance or impact on the network. Authors who are cited more often or are more influential may be represented by larger dots, while smaller dots indicate a lower frequency of joint citation. This type of network analysis can reveal patterns of collaboration, mutual influence, or areas of common interest between authors within the context of scientific research.

In this analysis, four clusters were identified. The first cluster, in red, includes 13 authors, and these researchers seek to emphasize the application of advanced technologies and analytics to improve dairy production and well-being, with a focus on monitoring and analytics technologies such as computer vision, sensors, and deep learning to monitor health, well-being, and livestock productivity. Another topic is automation and decision systems, such as automatic milking systems, automatic disease detection, and the use of models to support investment decisions. Finally, individualized management, monitoring of individual food consumption, and assessing body condition are important for optimizing production at the individual level. In other words, this cluster suggests much attention to articles that focus on the integration of emerging technologies in dairy farm management, with a strong component of technological innovation.

The second cluster, in green, includes 12 authors; in this cluster, some of the authors had articles found in this bibliometric research, as did the researcher of the first article found in this analysis, Matti Pastell. This group of researchers tends to focus more explicitly on the environmental behavior, welfare, and monitoring of animals, with an emphasis on animal behavior and welfare, monitoring, for example, the lying time and activities of animals and analyzing their welfare by sensors. Another topic is environmental and health activities and the use of a temperature–humidity health index to assess heat stress health monitoring based on behavior and environmental conditions. Some applications of these technologies include real-time positioning systems and accelerometers to monitor animals. This set indicates a greater production of articles focused on the interaction between the animal and its environment, with a particular emphasis on animal welfare and adaptation to environmental conditions.

At the intersection of technology and well-being in dairy farming, we observe duality, where the first group focuses on the drive for technological innovation, aiming to optimize production and management through the development and application of advanced technological solutions to meet the individual needs of animals, such as food and health. On the other hand, the second group focuses more directly on animal welfare and environmental behavior, with a special emphasis on adaptation, prioritizing understanding and improving the collective living conditions of the herd, and emphasizing general welfare and interaction with the environment. This duality reflects a trend in the field of dairy precision farming: while one side seeks productive efficiency through technological innovation, the other side highlights the critical importance of the practical application of these technologies to foster a healthy and sustainable environment for animals, showing that innovation and practical application are not mutually exclusive but rather complementary in promoting advancement in the dairy industry.

The third cluster, in blue, has six authors. Although the previous clusters also address technologies such as machine learning and computer vision, this cluster is notable for its particular focus on innovative and specific applications of these technologies. Unlike the previous clusters, which may include these technologies in a broader context of monitoring and managing animal health, the third cluster appears to more deeply explore the integration and specific application of advanced techniques to solve specific problems within dairy farming. This includes using models such as DeepLabCut for detailed behavioral analysis, applying inertial measurement units (IMUs) for more sophisticated behavior classification, and employing audio and video analytics for more comprehensive and detailed assessments of animal well-being. In summary, the notable difference of this cluster is in its targeted approach and detailed application of advanced technologies for specific and enhanced insights into animal behavior, health, and welfare, transcending the generic use of monitoring technologies to enter more specialized fields of behavioral analysis and automated diagnostics.

The last cluster, in yellow, has six authors; it particularly focuses on the welfare and behavioral management of animals, differentiating itself from the previous ones by its emphasis on the detailed monitoring of animal behavior to identify signs of stress, illness, and discomfort. This research group prioritizes the development and implementation of advanced technologies, such as wearable sensors and automated monitoring systems, to assess animal behavior and health in real time. One of the primary objectives is the early detection of adverse conditions, allowing for faster and more accurate interventions to improve animal welfare. Furthermore, there is a strong inclination toward behavioral analysis through techniques such as machine learning and computer vision, aiming to understand and promote management practices that respect the natural needs of animals. This approach not only raises welfare standards but also contributes to the sustainability and efficiency of livestock practices, offering insights for the continuous improvement of animal health and the environment in which they operate.

### 3.4. Most Influential Countries

**Figure 4:**
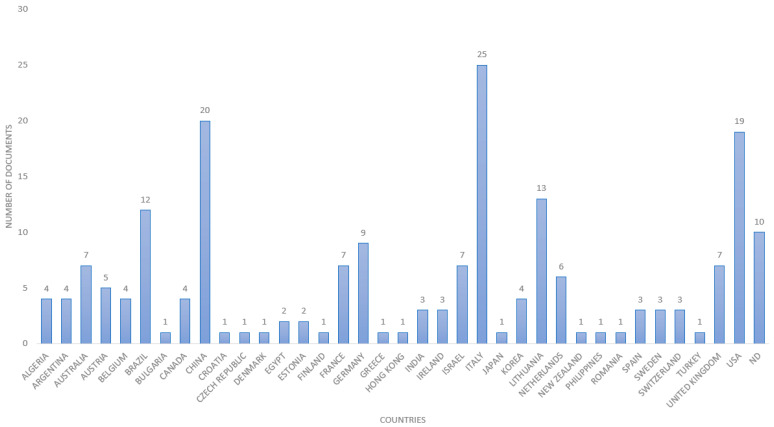
Number of documents by country.

The analysis of digital and precision livestock farming provides a comprehensive perspective on the global contributions to this ever-evolving research field. By scrutinizing the number of documents per country, as shown in Figure 4, an informative table provides insights into the most prominent nations in generating knowledge in this domain.

**Figure 5:**
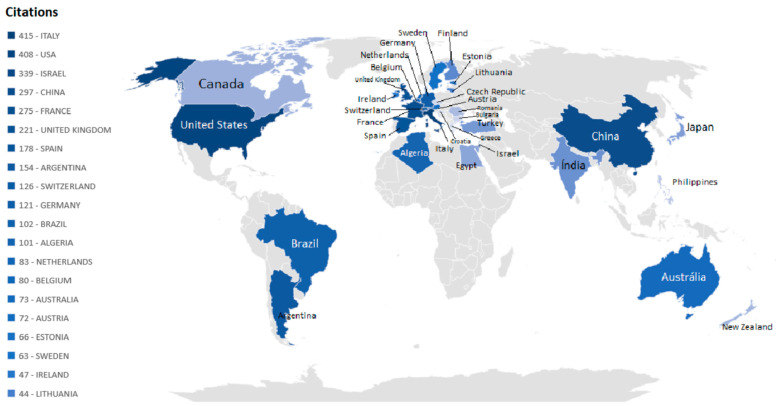
Number of citations by country.

Italy, China, and the United States of America (USA) are the largest producers of documents related to digital and precision livestock farming. Brazil is in fifth position, with 12 documents published, followed by Lithuania with 13 documents. However, the number of publications only assesses the productivity of a country, institution, or author, failing to reflect the importance or impact of the studies. In contrast, the number of citations measures the total impact [53]. Figure 5 displays a map of the number of citations per country for the top 20 countries with the highest number of citations.

Figure 4 and Figure 5 can be used to visualize the rankings of countries based on the number of documents and citations, facilitating the classification of the most relevant countries in this research field. Italy remains in the first position as the most relevant country, as it has not only the highest number of documents but also the highest number of citations (415 citations). China, with 97 citations, now occupies fourth place, while it occupied second place in the number of documents, being surpassed by the United States and Israel in the number of citations, with 408 and 339, respectively. Therefore, these countries are considered the most relevant and impactful in the field of digital and precision livestock farming. Brazil, with 12 documents identified, occupies the eleventh position in the ranking, with a total of 102 citations.

Countries such as China, the United States, and Italy are known for their advanced technological infrastructure. Access to innovative technologies and financial resources can boost research and the implementation of precision animal husbandry practices. Brazil is an agricultural and livestock powerhouse and one of the largest milk producers in the world. The economic relevance of the agricultural sector encourages investments in research and innovation.

### 3.5. Main Affiliations

The main research affiliations responsible for developing knowledge on the subject of digital and precision livestock in dairy cattle were identified (Table 3). The Lithuanian University of Health Sciences ranks highest, standing out as a prominent institution in research related to digital and precision livestock farming. Its leading position suggests a significant contribution to advancing knowledge in this specific field.

There is also notable influence from Italian institutions, with the presence of three (University of Catania, University of Bologna, and University of Padova), along with an institution from the United States (University of Kentucky) and one from the United Kingdom (University of Agricultural Engineering).

**Figure 6:**
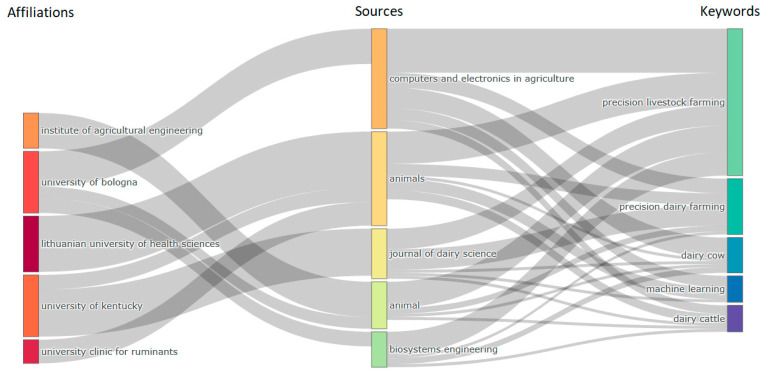
Diagram of main relationships between affiliations, sources/journals, and keywords.

In Bibliometrix, a map called a “Three-field Plot” is a visual tool that represents connections among three fields of analysis that can be selected within the research [28], and affiliations, sources, and keywords were selected for this analysis. Thicker links indicate stronger collaboration or more intense relationships between connected elements, and thinner links suggest less or less frequent collaboration. In this way, patterns in the sources that universities usually publish can be observed, providing a better understanding of each of the research fields and the key terms they use. Figure 6 presents this type of graph.

The connection between the Institute of Agricultural Engineering and the magazine “Animal”, which in turn connects with the five keywords “precision livestock farming”, “precision dairy farming”, “dairy cow”, “machine learning”, and “dairy cattle”, suggests a strategic and significant collaboration within the scope of digital and precision livestock farming. The Institute of Agricultural Engineering, by contributing issues to the journal “Animal”, demonstrates an interest in addressing broad, interdisciplinary issues related to animal production and welfare as covered by the journal. This collaboration may indicate expertise in engineering methods applied to agriculture and livestock, with a specific focus on the areas covered by the keywords.

By connecting with essential keywords for research, the magazine “Animal” positions itself as a crucial vehicle for disseminating research related to precision livestock farming. The strong presence of keywords, including “Machine Learning”, suggests a commitment to advanced, technologically oriented approaches to livestock management and animal production. This collaboration between the Institute of Agricultural Engineering and the magazine “Animal” reflects a strategic synergy between an academic institution and a specialized publication, reinforcing the importance of this partnership in promoting knowledge and innovations in precision livestock farming. Through this interaction, both entities contribute to advancing the field, offering a broad and technical view of the technologies that shape modern livestock farming.

The University of Bologna demonstrates a comprehensive approach to digital and precision livestock research by connecting with key journals. The close connection with the magazine “Animal” indicates a commitment to ethical and animal welfare issues, while the collaboration with “Biosystems Engineering” highlights its interest in applied engineering methods to agriculture. The connection with the magazine “Computers and Electronics in Agriculture” highlights its participation in the application of computational technologies in livestock management. These interactions highlight the diversity of the university’s contributions, reflecting its crucial role in forming knowledge and promoting innovative and sustainable practices in modern agriculture and livestock.

The Lithuanian University of Health Sciences and the University Clinic for Ruminants are heavily involved in the journal “Animals”, evidencing a central commitment to research on the welfare and behavior of ruminants in precision farming. The “Animals” magazine is a crucial platform for both institutions, covering essential topics for research, such as “Precision Livestock Farming”, “Precision Dairy Farming”, “Dairy Cow”, “Machine Learning”, and “Dairy Cattle”. This robust collaboration highlights the prominent role of these universities in producing knowledge aimed at technological and ethical advances in herd management and livestock practices, significantly contributing to the field of precision livestock farming.

Finally, the University of Kentucky maintains a substantial connection with the journal “Journal of Dairy Science” and a smaller connection with the journal “Animals”. The strong connection with the “Journal of Dairy Science” indicates a central commitment to biochemical, genetic, and public health research related to dairy production. This magazine is essential for understanding how precision farming impacts milk production and general practices in the dairy industry. The “Journal of Dairy Science” meaningfully connects to all keywords, highlighting the breadth of the University of Kentucky’s research in crucial areas, especially in “Precision Livestock Farming” and “Precision Dairy Farming”. This robust collaboration highlights the university’s prominent position in promoting knowledge and innovations in the field of precision livestock farming, with a specific focus on dairy production. The smaller connection with the magazine “Animals” suggests more selective participation that may be focused on ethical and animal welfare issues, contributing to a balanced approach to livestock research.

In general, all the analyzed universities and specialized journals establish prominent connections with the keyword “precision livestock farming”. This trend highlights the centrality of this topic in current precision livestock research. Furthermore, the five specific keywords (“precision livestock farming”, “precision dairy farming”, “dairy cow”, “machine learning”, and “dairy cattle”) emerge as interconnected elements, being approached in a varied and complementary way in several magazines. This scope reflects the multidisciplinary and interdisciplinary nature of precision farming, where technological advances, animal ethics, dairy production, and machine learning methods are intertwined to promote a comprehensive and innovative understanding of modern livestock management.

### 3.6. Keywords Related to Digital and Precision Livestock

**Figure 7:**
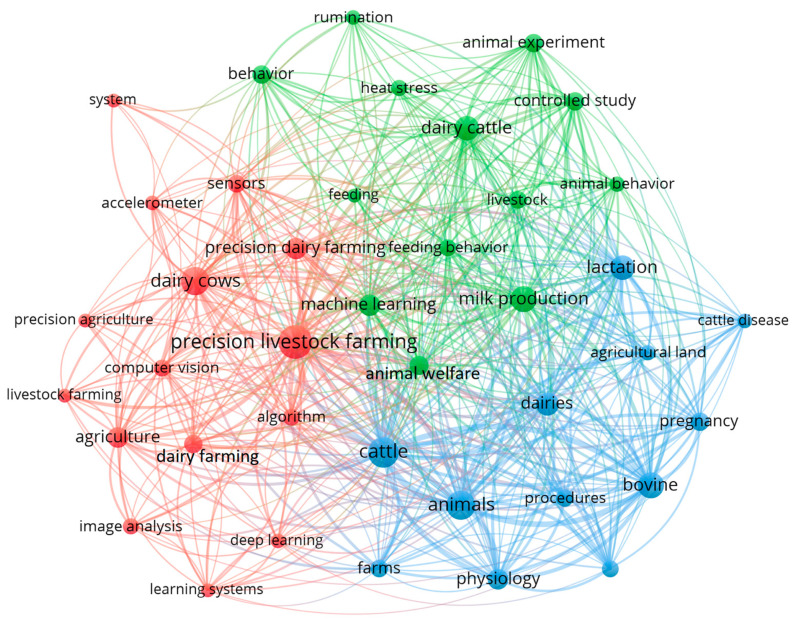
Network diagram displaying the interconnections between author keywords. The lines represent co-occurrences between the terms.

Examining keywords is a crucial approach for investigating the landscape of digital and precision livestock farming. The goal of this analysis is to examine the joint occurrence of specific terms associated with this domain, providing an in-depth understanding of prominent themes, patterns, and research areas. Exploring the co-occurrence of these keywords is helpful for identifying significant connections among various aspects within the context of digital and precision livestock farming. This analysis will contribute to a clearer delineation of key areas of interest and research directions in this continuously evolving field. The examination of keyword interrelations aims to pinpoint when these terms occur simultaneously in a specific sample, encompassing the title, abstract, or keyword list [31]. Figure 7 presents the co-occurrence analysis of author keywords in the analyzed documents.

In this analysis phase, several filters were applied to identify the most important keywords related to the studies. Initially, keywords with at least 10 occurrences were sought, resulting in the identification of 56 keywords in this process.

VOSviewer software cannot distinguish between words with different spellings or words in plural form compared to words in singular form. To address this issue, the software allows the optional inclusion of a “thesaurus file” in .txt format. Although optional, this file proves helpful in preventing the software from reading these words twice. Essentially, it combines them into a single entry, ensuring that they are interpreted only once. This practice prevents the generation of a map with duplicate words conveying the same meaning. When creating a map based on textual data, a VOSviewer thesaurus file can be used to group synonymous terms, account for different spellings and abbreviations with their full forms, or even disregard specific terms [30]. The adjusted and merged terms can be viewed in Table 4.

After this adjustment was made to combine synonyms and singular and plural forms, 46 words remained with a minimum of five occurrences for the analysis of the co-occurrence of keywords used in the studies. After the most relevant keywords were identified, additional filtering was performed to focus the analysis on specific connections related to the research topic. Consequently, words that did not directly contribute to the literature review, such as “article”, “eating”, “boss”, “female”, and others, were excluded. This refinement process aims to concentrate the analysis on interactions that are closely aligned with the scope of the research. The goal is to provide a more precise and focused examination of connections relevant to the context of digital and precision livestock farming.

Finally, 40 keywords meeting the criteria for this analysis were selected and divided into three clusters with different colors, where the analysis of the clusters reveals distinct trends and focuses on research related to digital and precision livestock farming.

The red cluster focuses on advanced technologies such as “sensors”, “algorithms”, “computer vision”, and “deep learning”. These technologies are fundamental to precision livestock farming, allowing the collection of detailed data on the behavior and conditions of animals, as well as optimizing agricultural processes. The presence of such terms as “agriculture” and “precision agriculture” strengthens the connection between these domains, indicating a holistic approach that incorporates precision agriculture strategies to optimize both agricultural and livestock production. This cluster reflects a promising synergy between digital livestock farming and advanced precision agriculture approaches, pointing to a future in which technology plays a central role in the efficiency and sustainability of integrated agricultural operations.

The green cluster addresses animal behavior, experiments, welfare, and nutrition, which are all crucial aspects of digital livestock farming. The inclusion of machine learning in this cluster highlights the importance of this technology in interpreting automated behavioral data and developing personalized feeding systems. Finally, the cluster in blue is closely linked to livestock farming, focusing on cattle, dairy cattle diseases, lactation, and reproductive health. Precision livestock farming uses technologies from this cluster to monitor animal health, optimize reproduction, and ensure efficient milk production on farms.

Despite being an advanced and current technology, the term “machine learning” is not located in the red cluster but in the green cluster; however, it is related to the other two clusters and directly related to the term “precision livestock farming”, highlighting its central position in precision livestock farming, connecting advanced data collection, animal behavior interpretation, and health and reproduction management. This centrality highlights the crucial role of machine learning by integrating different aspects of precision farming, making it a vital tool for modern agriculture and livestock.

The clusters are interconnected in precision livestock farming, represented as the largest circle in the diagram, as data collection with advanced technologies (red cluster) is essential for understanding and improving the behavior and well-being of animals (green cluster) while monitoring the health and productivity of dairy cattle (blue cluster).

The top five terms with the highest frequencies presented in this analysis were “precision livestock farming”, with 102 appearances, followed by the terms “cattle” (81 occurrences), “animals” (70 occurrences), “dairy cows” (67 occurrences), and “milk production”.

### 3.7. Trends in Research on Digital and Precision Livestock

Research has been developed in line with the availability and advancement of new technologies applied to dairy farming. To analyze trends in terms used in digital and precision livestock farming, an approach can be employed using the Bibliometrix “Trend Topics” chart [28], highlighting the 20 keywords of the most popular authors at specific times. By focusing on when a keyword emerges, discussing relevant work from that specific year, and understanding when its prominence diminishes, this method offers a dynamic visualization of research topics. This approach facilitates a gradual exploration of the temporal dynamics of key terms, enabling a more granular understanding of their trajectories and increasing prominence and subsequent relevance. The use of overlay and trending topic graphs provides a comprehensive overview, combining the temporal evolution of individual keywords with a snapshot of the most relevant terms at different times, enriching the analysis of digital and precision livestock literature.

**Figure 8:**
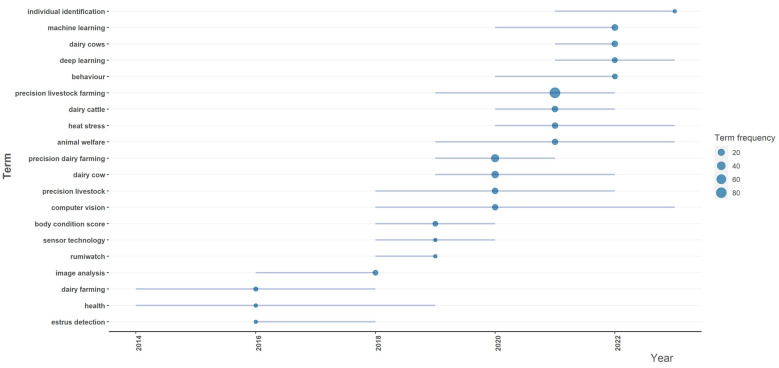
Trending words in Digital and Precision Livestock from 2014 to November 2023.

It was considered that the parameters for finding these trending words should have at least a frequency of three appearances and present up to four intervals per year. The terms used for this analysis can be found in Figure 8.

The expression “precision livestock farming” has shown impressive growth over the last few years, reaching a peak of 84 occurrences. This increase indicates a growing interest in and recognition of the importance of precision in livestock management.

In a sign of terminological evolution in the area of livestock farming, a significant change in the nomenclature associated with dairy production was observed after 2018. Although the term “dairy farming” was prominent until this period, as of 2019, there has been a transition to a more specific approach, reflected in the terms “dairy cow”, “dairy cattle”, and “dairy cows.” This shift suggests an increasing emphasis on the individualization and specificity of dairy cattle in their entirety. By focusing on more specific elements of milk production, such as the dairy cows themselves, the literature seems to direct its attention toward more specialized and personalized strategies, in line with the increasing implementation of precision technologies in livestock management. This change in terminology reflects a greater awareness of the importance of more specific and personalized approaches in modern livestock farming, aiming to optimize production and improve animal welfare. “Dairy cattle” and “Dairy cows” also highlight a consistent increase in technologies, indicating continued interest in their precise application in dairy production. This may be related to the search for greater efficiency, quality, and animal welfare on dairy farms.

The emphasis on “image analysis” and “computer vision” suggests a growing reliance on visual technologies to collect and interpret data. This is crucial for monitoring animal behavior, health, and body condition. The transition from “image analysis” to “computer vision” over time in precision livestock farming reflects a significant paradigm shift in the way visual data are processed and interpreted. Initially, the term “image analysis” suggested the application of traditional methods to extract information from captured images, implying a more manual and specific approach.

The introduction of the term “computer vision” from 2016 indicates an evolution toward more advanced and automated methods. Computer vision incorporates algorithms and machine learning techniques not only to analyze images but also to understand visual content in complex ways. This change suggests a search for more intelligent and efficient solutions in the interpretation of visual data in precision livestock farming.

The continued use of the term “computer vision” over subsequent years highlights the continued relevance and adoption of this approach. The use of advanced techniques, such as machine learning, facilitates a more sophisticated interpretation of images, offering detailed insights into animal behavior, health, and environmental conditions. Computer vision is fundamental for identifying patterns, detecting anomalies, and customizing management strategies, thus contributing to the efficiency and sustainability of precision livestock farming. This transition reflects the constant search for innovations and improvements in the application of visual technologies in modern livestock management.

Currently, “machine learning” continues to be a fundamental piece, indicating the accurate application of algorithms that allow systems and software to learn patterns from data, enabling more predictions and decision-making. On the other hand, “deep learning” highlights the application of deep neural networks, enabling deeper and more complex data analysis, especially in vast and heterogeneous datasets.

These trends reflect the current emphasis in the industry on incorporating more advanced machine learning approaches to drive precision farming. The use of these technologies facilitates more sophisticated analysis of large datasets, helping to improve operational efficiency, optimize livestock management, predict health conditions, and promote sustainable agricultural practices.

Works related to “machine learning” and “deep learning” in the context of digital and precision livestock farming for dairy cattle are considered crucial at the moment, as they represent the latest frontiers of technological innovation in agriculture. The learning capabilities of these models contribute to a more personalized approach to livestock management, promoting more efficient and sustainable production. Therefore, continued investment and research in these areas are critical to driving precision farming into the future, aligning with growing demands for smarter, data-driven agricultural practices.

## 4. Limitations and Challenges of a Bibliometric Analysis

Bibliometric analysis is a method that allows researchers to explore and analyze large volumes of scientific data and to uncover the evolutionary nuances of a specific field while also elucidating emerging areas in that field [54]. Therefore, conducting a bibliometric analysis may present limitations due to the complexity of dealing with a large set of data. The vast amount of information contained in scientific records can introduce challenges in the appropriate interpretation and selection of data, highlighting the importance of careful methodological approaches and attention to the specific nuances of each dataset.

This study has several limitations that must be considered when interpreting its results. First, the choice to only consider works written in English may have introduced a linguistic limitation since there were analyses in other languages. This decision may have influenced the overall representativeness of the study, as relevant research in other languages may not have been included.

Another limitation lies in the approach taken for selecting papers based on keywords. The decision to include only those papers containing the specified keywords in the title, abstract, and keywords may have excluded valuable contributions that included these terms in other sections of the document, impacting the scope of the analysis. However, it underscores the importance of authors carefully choosing keywords and crafting precise abstracts. This practice is crucial for ensuring that essential elements such as keywords, abstracts, and titles accurately communicate the content of papers, overcoming limitations imposed by specific selection criteria, and contributing to the quality and comprehensiveness of bibliometric analyses.

Furthermore, it is important to recognize that the quality of bibliometrics significantly depends on adequate data. In the case of this study, the need to ensure uniformity in the data obtained prior to analysis was highlighted as a critical consideration. The presence of formatting errors or deviations in data, especially in spreadsheets used in bibliometric software such as Bibliometrix and VOSviewer, can affect the accuracy and reliability of interpretations. Thus, meticulous data standardization was an essential step to ensure the validity of the conclusions presented in this work.

## 5. Conclusions

In conclusion, the bibliometric analysis revealed a clear emphasis by researchers on technologies, especially computer vision and machine learning in dairy cattle farming, which have been inserted into the field in conjunction with the temporal evolution of research. A significant increase in scientific publications by universities was identified, in addition to a large participation by countries through citations of works on this research topic.

The main universities that were identified as having a large share of documents were the “Lithuanian University of Health Sciences”, “University of Kentucky”, “Institute of Agricultural Engineering”, and some Italian universities, such as “University of Catania”, “University of Bologna”, and “University of Padova”. The main countries ranked by number of citations were Italy, the USA, Israel, and China.

In the context of this bibliometric analysis, it is important to mention the participation of Brazil, which had 12 documents and was ranked 11th in terms of the number of citations. Although this position may reflect its significant presence in dairy cattle research, it is crucial to note that, compared to other countries, there is scope for growth in scientific contributions. A more critical analysis reveals the opportunity to boost knowledge production through investments in emerging technologies, aligning with global trends in computer vision, machine learning, and precision livestock farming. Brazil, with its vast experience in the agricultural sector, has the potential to play a crucial role in the adoption and advancement of these technologies, contributing not only to sustainable dairy production but also to the consolidation of a prominent position on the international scientific scene.

Finally, the work reflected current research trends and demands, especially with regard to behavioral monitoring and management of heat stress to promote animal welfare; that is, research points to the need for the use of current technologies to improve management and productivity in dairy cattle farming. The increasing use of these technologies not only boosts operational efficiency but also responds to contemporary demands for sustainable and ethical practices in dairy production. The constant evolution of these approaches suggests a significant transition toward smarter, animal-centred management systems. Therefore, research emphasizes the imperative need to integrate advanced technologies in precision livestock farming, paving the way for more efficient, responsible dairy production adapted to the challenges of the 21st century.

## Figures and Tables

**Table 1:** Top 10 scientific publications on digital and precision livestock from 2008 to November 2023, ranked by citation number.

R	Title	Authors	PY	Journal	NC
1	Classification of behavior in housed dairy cows using an accelerometer-based activity monitoring system	Vázquez et al. [35]	2015	Animal Biotelemetry	166
2	An intelligent Edge-IoT platform for monitoring livestock and crops in a dairy farming scenario	Alonso et al. [36]	2020	Ad Hoc Network	163
3	Smart Animal Agriculture: Application of Real-Time Sensors to Improve Animal Well-Being and Production	Halachmi et al. [4]	2019	Annual Reviews	114
4	System specification and validation of a noseband pressure sensor for measurement of ruminating and eating behavior in stable-fed cows	Zehner et al. [37]	2017	Computers and Electronics in Agriculture	108
5	Development of automatic body condition scoring using a low-cost 3-dimensional Kinect camera	Spoliansky et al. [38]	2016	Journal of Dairy Science	94
6	Influence of milk yield, stage of lactation, and body condition on dairy cattle lying behavior measured using an automated activity monitoring sensor	Bewley et al. [33]	2010	Journal of Dairy Research	89
7	Computer vision system for measuring individual cow feed intake using RGB-D camera and deep learning algorithms	Bezen et al. [39]	2020	Journal of Dairy Science	70
8	The automatic detection of dairy cow feeding and standing behaviors in free-stall barns by a computer vision-based system	Porto et al. [40]	2015	Biosystems Engineering	69
9	Behavioral and physiological changes around estrus events identified using multiple automated monitoring technologies	Dolecheck et al. [41]	2015	Journal of Dairy Science	69
10	Image analysis for individual identification and feeding behavior monitoring of dairy cows based on convolutional neural network (CNN)	Achour et al. [42]	2020	Biosystems Engineering	64

R: ranking; PY: publication year; NC: number of citations.

**Table 2:** Top 8 sources of publications in the world on digital and precision livestock from 2008 to November 2023.

Journal	SJR ^1^	CiteScore ^2^	JCR ^3^	H-i	ISSN	ND	NC
Computers and Electronics in Agriculture [44]	1.587	13.6	8.3	149	0168-1699	33	872
Journal of Dairy Science [45]	1.179	7.4	3.5	216	0022-0302	23	715
Biosystems Engineering [46]	1.061	10.1	5.1	125	1537-5110	11	311
Animals [47]	0.684	4.2	3.0	60	2076-2615	25	225
Animal [48]	0.902	6.6	3.6	91	1751-7311	12	196
Animal Biotelemetry [49]	0.813	4.2	2.7	29	2050-3385	1	166
Ad Hoc Networks [50]	1.301	12.1	4.8	104	1570-8705	1	163
Journal of Dairy Research [51]	0.465	3.5	2.1	84	1469-7629	5	122

^1^ SJR (SCImago Journal Rank): Web of Science Index; ^2^ CiteScore: Scopus Index; ^3^ JCR (Journal Citation Reports—Journal Impact Factor): Scopus Index; H-i: H Index; ND: Number of documents and NC: Number of citations.

**Table 3:** Top 6 main research institutions in the research field of digital and precision livestock in dairy farms from 2008 to November 2023.

R	Organizations	Countries	ND
1°	Lithuanian University of Health Sciences	Lithuania	13
2°	University of Kentucky	United States of America	7
3°	Institute of Agricultural Engineering	United Kingdom	5
4°	University of Catania	Italy	5
5°	University of Bologna	Italy	4
6°	University of Padova	Italy	4

**Table 4:** Dictionary of synonyms based on keywords.

Synonyms Considered	Merge of Terms
animals; animal; animalia	animals
cattle diseases; cattle disease	cattle diseases
dairy cow; dairy cows; dairy-cows	dairy cows
dairies; dairying	dairies
milk production; milk; milk yield	milk production
physiologic monitoring; monitoring, physiologic	physiologic monitoring
precision livestock farming; precision livestock	precision livestock farming
sensors; sensor	sensors

## Data Availability

The original contributions presented in the study are included in the article.
